# The Abnormality of Topological Asymmetry in Hemispheric Brain Anatomical Networks in Bipolar Disorder

**DOI:** 10.3389/fnins.2018.00618

**Published:** 2018-09-03

**Authors:** Bin Wang, Ting Li, Mengni Zhou, Shuo Zhao, Yan Niu, Xin Wang, Ting Yan, Rui Cao, Jie Xiang, Dandan Li

**Affiliations:** ^1^College of Information and Computer, Taiyuan University of Technology, Taiyuan, China; ^2^Faculty of Human Health Science, Graduate School of Medicine, Kyoto University, Kyoto, Japan; ^3^Translational Medicine Research Center, Shanxi Medical University, Taiyuan, China

**Keywords:** bipolar disorder, diffusion tensor imaging, graph theory, hemispheric asymmetry, structural connectivity

## Abstract

Convergent evidences have demonstrated a variety of regional abnormalities of asymmetry in bipolar disorder (BD). However, little is known about the alterations in hemispheric topological asymmetries. In this study, we used diffusion tensor imaging to construct the hemispheric brain anatomical network of 49 patients with BD and 61 matched normal controls. Graph theory was then applied to quantify topological properties of the hemispheric networks. Although small-world properties were preserved in the hemispheric networks of BD, the degrees of the asymmetry in global efficiency, characteristic path length, and small-world property were significantly decreased. More changes in topological properties of the right hemisphere than those of left hemisphere were found in patients compared with normal controls. Consistent with such changes, the nodal efficiency in patients with BD also showed less rightward asymmetry mainly in the frontal, occipital, parietal, and temporal lobes. In contrast to leftward asymmetry, significant rightward asymmetry was found in supplementary motor area of BD, and attributed to more deficits in nodal efficiency of the left hemisphere. Finally, these asymmetry score of nodal efficiency in the inferior parietal lobule and rolandic operculum were significantly associated with symptom severity of BD. Our results suggested that abnormal hemispheric asymmetries in brain anatomical networks were associated with aberrant neurodevelopment, and providing insights into the potential neural biomarkers of BD by measuring the topological asymmetry in hemispheric brain anatomical networks.

## Introduction

The human brain is structurally and functionally asymmetrical or lateralized (Watkins et al., 2001; Toga and Thompson, 2003). Even subtle perturbations to anatomical asymmetries between two hemispheres, such as gray matter volume (Sigalovsky et al., 2006), cortical thickness (Rimol et al., 2010), or white matter (WM) integrity (Park et al., 2004), can cause disturbances in cognitive and emotion processes. Studies have shown that aberrant brain region asymmetries are highly correlated with disturbed functions such as executive function (Yin et al., 2013), emotion (Schulte et al., 2012), and language (O’Donoghue et al., 2017). Hence, abnormal anatomical asymmetries have been observed in a variety of neurological and psychiatric disorders, including schizophrenia (Highley et al., 1999), depression (Kwon et al., 1996), attention-deficit hyperactivity disorder (Miller et al., 2006), and Bipolar disorder (BD) (Beyer et al., 2005; Bruno et al., 2008). As one of the most distinct syndromes in psychiatry, BD is mainly characterized as episodic elevations in emotion and disturbances in cognition (Belmaker, 2004; Saunders and Goodwin, 2010). Convergent studies on BD have showed abnormal asymmetries in WM (Bruno et al., 2008; Kafantaris et al., 2009; Wessa et al., 2009; Mahon et al., 2013), for example, less WM volume within the left frontal lobes, the rightward WM in orbital frontal. These results indicated that the alteration in WM asymmetries have been proposed as a key factor in the manifestation of BD symptoms.

As an imaging method, diffusion tensor imaging (DTI) can reconstruct the major WM tracts faithfully (Dae-Jin et al., 2013) and has been proved to be a promising tool for assessing WM abnormalities. Recently, using the DTI tractography and graph theory, connectome studies demonstrated abnormal topological properties in BD (Leow et al., 2013; Forde et al., 2015; Collin et al., 2016, 2017; Spielberg et al., 2016; O’Donoghue et al., 2017). The patients with BD showed decrease in global integration (longer characteristic path length, smaller global efficiency), increase in functional segregation (larger clustering coefficient and local efficiency), and loss of small-world property (the balance between local integration and functional segregation) (Leow et al., 2013; Forde et al., 2015; Collin et al., 2016; Spielberg et al., 2016; O’Donoghue et al., 2017). These studies have mainly focused on the WM topological properties in whole network rather than hemispheric network. Analyzing the hemispheric anatomical networks and further determination of the status of the anatomical network asymmetries might benefit the understanding of the underlying nature of alteration in the brain of BD, and potentially help to elucidate the etiology of the disorder. However, the hemispheric asymmetries of anatomical network in patients with BD were remained unclear.

In present study, we adopted the DTI deterministic tractography method and graph theory to investigate the abnormality of hemispheric asymmetries in brain anatomical networks in BD. In particular, we focused on global graph measures, including small-world property, global and local efficiency, and regional parameters to evaluate (1) the abnormal hemispheric asymmetries in brain anatomical networks in patients with BD and (2) whether the abnormal hemispheric asymmetries in network organization were related to clinical features of BD.

## Materials and Methods

### Subjects

Data were selected from the UCLA Consortium for Neuropsychiatric Phenomics LA5c Study, and the study was approved by the UCLA Institutional Review Board. The data were obtained via a public database, openfMRI (Poldrack and Gorgolewski, 2015). About 49 BD patients and 61 age- and gender-matched normal subjects were selected for further analyzing. All subjects were right-handed. There are more details available in openfMRI^1^ (ds000030). The detailed demographics and clinical features of the patients with BD and normal controls are described in **Table 1**. Patient symptoms were evaluated using the 17-item Hamilton Depression Rating Scale (HAMD) (Hamilton, 1960) and the Young Mania Rating Scale (YMRS) (Young et al., 1978).

**Table 1:** Demographic and clinical characteristics^a^.

Characteristic	Group (patients/controls = 49/61)		Statistical test	
	Patients with BD	Normal controls		
Age (years)	22–50(32.3 ± 9.0)	21–49(33.1 ± 9.2)	*t*_108_ = -1.218	*P* = 0.226^b^
Male/Female	28/21	32/29	χ_1_^2^ = 0.909	*P* = 0.340^c^
Education (years)	11–19(14.6 ± 2.0)	12–19(15.2 ± 1.5)	*t*_108_ = -2.080	*P* = 0.040^b^
Duration of illness (months)	0–24(2.1 ± 5.2)	N/A		
Medication dose (mg/day)	0–6210(784.8 ± 1035.3)			
Handscore^d^	0.75–1(0.93 ± 0.1)	0.80–1(0.93 ± 0.1)		
YMRS_score^e^	0–41(11.9 ± 11.0)	N/A		
HAMD^e^	0–32 (12.0 ± 8.4)	N/A		

*^a^Unless otherwise indicated, data are expressed as a range of minimum–maximum (mean ± SD). ^b^The *P*-value was obtained using a two-sample two-tailed t-test. ^c^The P-value was obtained using a two-tail Pearson’s χ^2^ test. ^d^The Handscore described the handedness of subjects. It was obtained using a formula (Right - Left)/(Right ± Left). ^e^The score of Young Mania Rating Scale (YMRS_score) and the 17-item Hamilton Depression Rating Scale (HAMD) was used to assess the symptom severity of patients with BD.*

### Data Acquisition and Preprocessing

Structural MRI data were acquired on 3T Siemens Trio scanners located at the Ahmanson-Lovelace Brain Mapping Center (Siemens version syngo MRB15) and the Staglin Center for Cognitive Neuroscience (Siemens version syngo MRB17) at UCLA. A high-resolution 3D echoplanar imaging was acquired with the following parameters: TR = 1.9 s, TE = 2.26 ms, FOV = 250 mm, matrix = 256 × 256, sagittal plane, slice thickness = 1 mm, 176 slices. Diffusion weighted imaging (DWI) data were collected using an echo-planar sequence with parameters: 64 directions, 2 mm slices, TR/TE = 9000/93 ms, 1 average, 96 × 96 matrix, 90° flip angle, axial slices, *b* = 1000 s/mm^2^.

This study used the MATLAB toolbox named PANDA to perform data preprocessing and the construction of the brain network (Cui et al., 2013). Specifically, data preprocessing approaches included correction for simple head movements and eddy current distortions using affine transformation to the b0 image (Jenkinson et al., 2002). After data preprocessing, the seven independent components of the diffusion tensor were estimated and from which fractional anisotropy (FA, a DTI measurement) was calculated. Subsequently, the deterministic fiber tracking algorithms were used to reconstruct fiber paths (Mori et al., 1999). The fiber tracking procedure started from the deep WM regions and terminated if two consecutive moving directions had a crossing angle above 35° or the FA was out of the threshold range (0.1∼1).

### Network Construction and Analysis

In this study, the method of constructing the WM network was described in Gong et al. (Gong et al., 2009). Based on the automated anatomical labeling (AAL) parcellation scheme (Tzouriomazoyer et al., 2002), the brain was divided into 90 regions (45 in each hemisphere). Each region was defined as one node in the anatomical network. A linear transformation was applied locally within each subject’s DTI image correlated with the T1-weighted image to coregister them to the b0 image with DTI space followed by applying a nonlinear transformation to map to the ICBM152 T1 template [Montreal Neurological Institute (MNI)]. Then, the subject-specific AAL mask was weaved from the MNI space to the DTI native space with the corresponding inverse transformation, such that separate labeling values were maintained via nearest-neighbor interpolation (Gong et al., 2009; Cui et al., 2013). The FA between two regions was defined as the network edge (Shu et al., 2011; Bai et al., 2012). Prior to constructing the network, the connection between these two regions was adopted if the fiber number (FN) between the two regions was larger than 3 (Shu et al., 2011). It was helpful to reduce the influence of pseudo-connections owing to possible noise effects on whole-brain tractography. For each subject, we eliminated the inter-hemispheric connections and then obtained two weighted 45 × 45 hemispheric brain networks, one for the left hemisphere and the other for the right hemisphere.

The network architecture was then investigated at both global and regional levels for the constructed WM networks. The small-world property suggests the architecture of networks with higher local clustering and equivalent characteristic path length compared with the random network (Watts and Strogatz, 1998). In this work, eight network properties were used to analyze the topological organization of the WM networks. The clustering coefficient (C_p_) of a network is thus the average of clustering coefficients across nodes and is a measure of functional segregation. The characteristic path length (L_p_) of a network is the average shortest path length between all pairs of nodes in the network and is the most commonly used measure of global integration. The normalized clustering coefficients (γ), γ = C_p_/C_rand_ and the normalized characteristic path length (λ), λ = L_p_/L_rand_, C_rand_ and L_rand_ represent indices derived from matched random networks (100 matched random networks were selected). The small-world property of a network can be characterized by both γ and λ, indicating a balance between integration and segregation. In a small-world network, the C_p_ is significantly higher than that of random networks (γ greater than 1), while the L_p_ is comparable to random networks (λ close to 1). The global efficiency (E_g_), reflecting the efficiency of whole network information transmission (integration), is the inverse relation of L_p_. The local efficiency (E_loc_), reflected the efficiency of the network segregation. Regional properties were described in terms of nodal efficiency, E_nodal_(*i*) (Achard and Bullmore, 2007). It measures the information transmission ability of node *i* in the network. A node with high E_nodal_(*i*) indicates great interconnectivity with other regions in the network. The detailed descriptions of these graph theory parameters can be found in a previous work (Bullmore and Sporns, 2009; Rubinov and Sporns, 2010). The graph theory analysis was performed with GRETNA^2^ software.

### Asymmetry Score

The degree of hemispheric network asymmetry was estimated by the asymmetry score (Iturriamedina et al., 2011; Ratnarajah et al., 2013) using the following formulation: AS(X) = 100 × [X(R) − X(L)]/[X(R) + X(L)], where X(R) and X(L) represent the network properties of the right and left hemisphere, respectively. The asymmetry score AS(X) helps us look at the differences between the right and left hemisphere. Notably, a positive value of AS(X) represents a rightward asymmetry, while a negative value of AS(X) indicates a leftward asymmetry.

### Statistical Analysis

All statistical analyses were performed using SPSS 19 software (SPSS, Inc., Chicago, IL, United States). To determine whether there was any significant group difference in age and education, this work performed the separate two-tailed *t*-tests to analyze data. We used a χ^2^ test to analyze the gender data. To assess the group differences in hemispheric network properties, we used a general linear model (GLM) that was performed with hemisphere as a within-subject factor, group as a between-subject factor, and a group-by-hemisphere interaction. Studies have shown that hemispheric asymmetries are related to age (Cabeza, 2002; Dolcos et al., 2002) and gender (McGlone, 1980; Good et al., 2001). Hence, we set the age, gender, and age-by-gender interaction as covariates in the GLM. Further *post hoc* tests, including two-sample *t*-test for group differences and paired *t*-test for hemisphere differences, were used if any difference survived a threshold of *P* < 0.05. To determine whether the *AS* of the network properties within each group was significantly different from zero, one-sample *t*-test was performed on the asymmetry score. Meanwhile, a univariate analysis with covariance of age, gender, and age-by-gender interaction was performed on the *AS* to assess group differences. The threshold of *P* < 0.05 was considered to be significant for global properties. In particular, for the regional properties, the Bonferroni-correction was performed on that threshold (*P* < 0.05).

This work also studied the relationships between the hemispheric asymmetry scores and symptom severity of patients with BD. Considering age, gender, and age-by-gender as the covariates, we used both Pearson and Spearman correlation to analyze the relationship between network properties and BD symptom severity. Pearson is used to measure the linear relationship between two consecutive variables. Spearman does not require the distribution of primitive variables and belongs to a non-parametric statistical method. It was unnecessary to correct multiple comparisons because the aforementioned analyses were exploratory in nature. Hence, a significant relationship was considered at an uncorrected *P*-value of 0.05.

## Results

### Global Properties of Hemispheric Networks

#### Group and Hemispheric Differences

**FIGURE 1:**
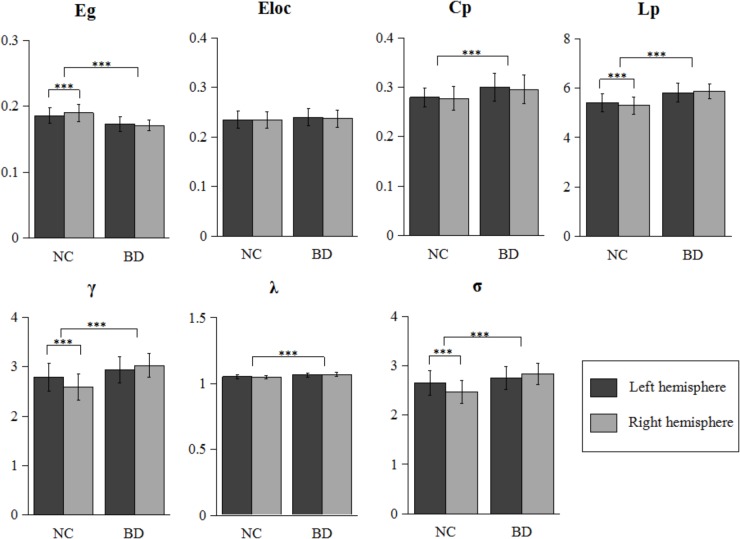
Further statistical analysis of network properties. Bars represent mean ± SD. Each horizontal line and associated number represent the *P*-value of a *t*-test (paired *t*-test for hemispheric difference at each group and two-sample *t*-test for group difference). Significant differences are marked with asterisk (^∗∗∗^*P* < 0.001). BD, bipolar disorder; NC, normal control.

As shown in **Figure 1**, all four hemispheric brain anatomical networks (2 hemispheres × 2 groups) exhibited prominent features of small-world property, as expressed by a larger γ (γ > 1) and a smaller λ (λ ≈ 1). Statistical analysis results showed significantly topological changes in the global properties in both groups and hemispheres. Except for local efficiency E_loc_ (*F*_1,106_ = 2.372, *P* = 0.127), the rest of the six network properties exhibited significant group differences between normal controls and patients with BD. There was a reduced global integration (decreased global efficiency and increased characteristic path length) and increased small-world property in patients with BD. Furthermore, we observed a significant group-by-hemisphere interaction on global efficiency E_g_ (*F*_1,106_ = 9.311, *P* = 0.003), the characteristic path length L_p_ (*F*_1,106_ = 7.323, *P* = 0.008), the normalized clustering coefficients γ (*F*_1,106_ = 30.107, *P* < 0.001) and the small-world property σ (*F*_1,106_ = 32.163, *P* < 0.001). *Post hoc* analysis indicated that this interaction resulted from a significant rightward trend in global integration (*P* = 0.001) and a significant leftward trend in the characteristic path length (*P* = 0.003), the normalized clustering coefficients (*P* < 0.001), and small-world architecture (*P* < 0.001) in normal controls and a symmetrical trend in patients with BD.

#### Asymmetry Score

The asymmetry score was helpful for us to evaluate the differences between the right and left hemisphere for the network properties. Additionally, the group differences in asymmetry scores would directly reflect the abnormal of hemispheric lateralization of topological organization in BD, and supplemented the result of the group-by-hemisphere interaction. **Table 2** summarizes the statistical analysis results of the asymmetry scores of the global network properties for the two groups. Significant differences in hemispheric asymmetry were only observed in normal controls and disappeared in patients with BD. Normal controls showed more globally efficient in the right hemisphere than the left hemisphere [AS(E_g_), *t*_52_ = 3.852, *P* < 0.001]. Additionally, the characteristic path length [AS(L_p_), *t*_52_ = -3.852, *P* < 0.001], the normalized clustering coefficients [AS(γ), *t*_52_ = -6.447, *P* < 0.001], and the small-world property [AS(σ), *t*_52_ = -6.578, *P* < 0.001] showed leftward hemispheric asymmetries in normal controls. When comparing the asymmetry scores between two groups, we observed significant group differences in asymmetry scores of E_g_ (*F*_1,106_ = 8.268, *P* = 0.005), L_p_ (*F*_1,106_ = 8.268, *P* = 0.005), γ (*F*_1,106_ = 33.684, *P* < 0.001), and σ (*F*_1,106_ = 35.657, *P* < 0.001), which agreed with the significant group-by-hemisphere interaction on E_g_, L_p_, γ, and σ revealed by the GLM model. This result indicated that the rightward asymmetries of global integration and leftward asymmetries of small-world feature were observed only in normal controls but disappeared in patients with BD.

**Table 2:** Group differences on the asymmetry scores of the network properties.

Properties	Patients with BD *t*_48_ (*P*-value)	NC subjects *t*_60_ (*P*-value)	BD versus NC *F*_1,106_ (*P*-value)
AS(E_g_)	-0.201(0.842)	3.852( **< 0.001**)	8.268(**0.005**)
AS(E_loc_)	-0.756(0.453)	0.078(0.938)	0.048(0.827)
AS(C_p_)	-0.719(0.476)	-0.591(0.557)	0.001(0.998)
AS(L_p_)	1.118(0.269)	-3.852( **< 0.001**)	8.268(**0.005**)
AS(γ)	0.916(0.061)	-6.447( **< 0.001**)	33.684( **< 0.001**)
AS(λ)	0.693(0.491)	-1.624(0.110)	1.649(0.202)
AS(σ)	1.899(0.064)	-6.578( **< 0.001**)	35.657( **< 0.001**)


*A one-sample two-tailed t-test was used to evaluate the statistical results within each group. The between-group differences were computed via a univariate ANCOVA, and the effects of age, gender, and age by-gender interaction were controlled for all of these analyses. A negative t-value within each group shows a leftward asymmetry and vice versa. The significant effect (P < 0.05) of network property was expressed in bold. BD, bipolar disorder; NC, normal control.*

### Regional Properties of the Hemispheric Networks

#### Hemispheric and Group Differences

**FIGURE 2:**
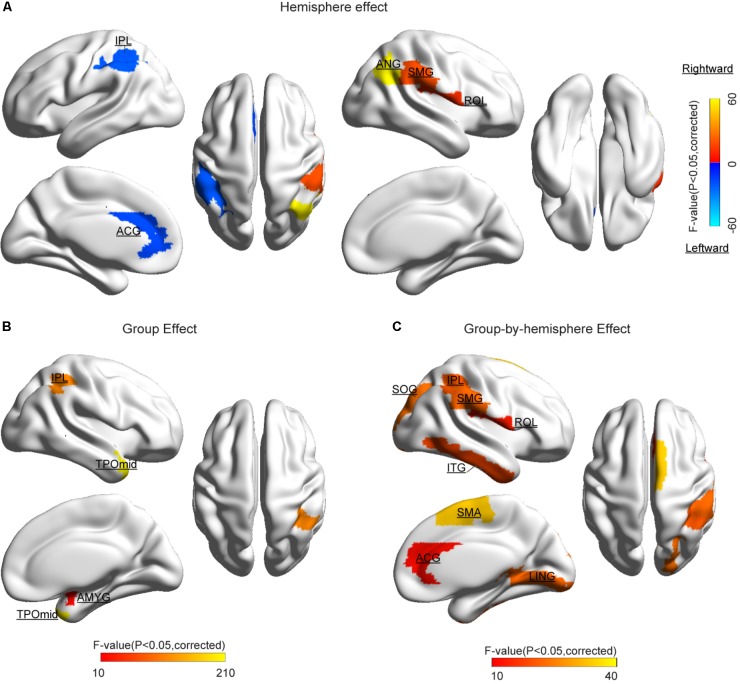
Cortical regions with significant differences **(A)** hemispheric difference, **(B)** group difference, and **(C)** group-by-hemispheric interaction on the nodal efficiency. The color bar denotes *F*-values. Significant difference was identified with a threshold value of *P* < 0.05 (Bonferroni-corrected). Significantly different regions were overlaid on surface maps provided by BrainNet Viewer software (Xia et al., 2013).

**FIGURE 3:**
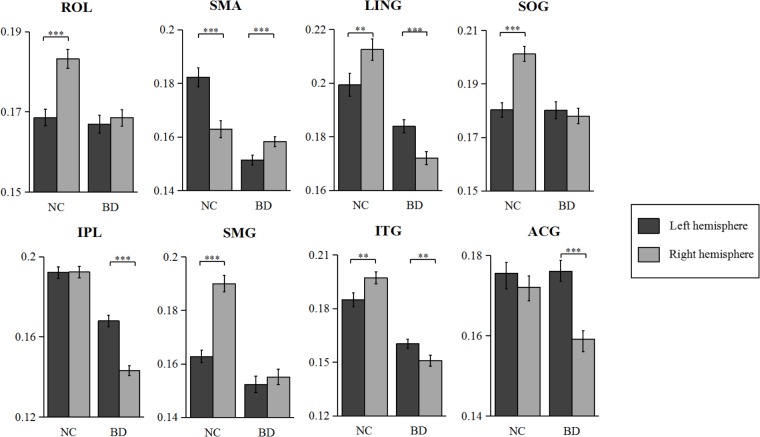
The nodal efficiency of normal controls and bipolar disorder in several regions including PUT, ROL, and SFGdor. Error bar indicates standard error. ^∗∗∗^*P* < 0.001, ^∗∗^*P* < 0.01.

The statistical results of nodal efficiency differences are summarized in **Figure 2**. Using Bonferroni-correction, we observed five regions exhibited significant hemispheric differences (*P* < 0.05) (**Figure 2A**). Among these five brain regions, regions with significant leftward advantage in nodal efficiency mainly included the anterior cingulate gyrus (ACG) and the inferior parietal lobule (IPL), whereas regions with significant rightward nodal efficiency were predominantly located at the supramarginal gyrus (SMG), the angular gyrus (ANG), and the rolandic operculum (ROL). Moreover, regions with significant group differences (BD < CN, *P* < 0.05, Bonferroni-corrected) in nodal efficiency included the amygdala (AMYG), IPL, putamen (PUT), and temporal pole (middle) (TPOmid) (**Figure 2B**). The PUT region could not be marked in **Figure 2B**. Furthermore, we found significant group-by-hemisphere interactions in the ROL (*F*_1,106_ = 12.930, *P* < 0.001), the lingual gyrus (LING) (*F*_1,106_ = 19.011, *P* < 0.001), the superior occipital gyrus (SOG) (*F*_1,106_ = 21.221, *P* < 0.001), the IPL (*F*_1,106_ = 36.225, *P* < 0.001), the SMG (*F*_1,106_ = 21.617, *P* < 0.001), the inferior temporal gyrus (ITG) (*F*_1,106_ = 15.900, *P* < 0.001), and the ACG (*F*_1,106_ = 10.547, *P* = 0.002, **Figure 2C**). *Post hoc* analysis indicated that these interaction effects resulted from significantly reduced rightward hemispheric asymmetry in nodal efficiency in patients with BD (**Figure 3**). Specially, we revealed significant group-by-hemisphere interaction in the supplementary motor area (SMA) (*F*_1,106_ = 34.907, *P* < 0.001). *Post hoc* analysis showed that this interaction in SMA was attributed to significantly rightward hemispheric asymmetry in nodal efficiency in patients with BD (**Figure 3**).

#### Asymmetry Score

**FIGURE 4:**
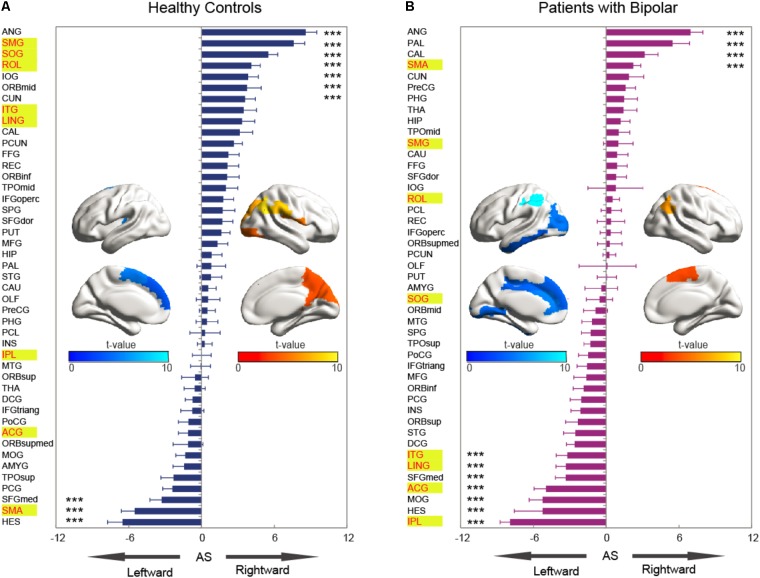
The asymmetry scores of nodal efficiency for the hemispheric network with 45 nodes. Each bar indicates the mean asymmetry scores (AS). Error bars represent standard error. For both groups of **(A)** normal controls and **(B)** patients with BD, regions with significant AS are marked with asterisk (^∗∗∗^*P* < 0.001, Bonferroni-corrected). All the regions in each group were ascending sorted according to the statistical *t*-values. Similarly, the color bar indicated the statistical *t*-values. The spatial distributions of cortical regions with significant AS in both groups were also presented with BrainNet Viewer software (Xia et al., 2013). Cortical regions with significant group difference were presented with yellow background.

The asymmetry score of nodal efficiency indicated that the region was leftward or rightward in each group subject. Consistent with the prior GLM hemispheric results, both groups exhibited significant hemispheric asymmetry (*P* < 0.05, Bonferroni-corrected) in nodal efficiency. For the normal controls shown in **Figure 4A**, the nodal efficiency with rightward asymmetry covered the inferior occipital gyrus (IOG), the orbitofrontal gyrus, the middle part (ORBmid), Cuneus (CUN), and ANG, SMG, SOG, and ROL. In contrast, the leftward asymmetric nodes were mainly located at the superior frontal gyrus, medial part (SFGmed), SMA, and Heschl (HES). For patients with BD shown in **Figure 4B**, the regions with significant leftward asymmetries in nodal efficiency involved the ITG, LING, SFGmed, ACG, middle occipital gyrus (MOG), HES, and IPL regions. Regions with significant rightward asymmetries in nodal efficiency were located at the ANG, Pallidium (PAL), CAL, and SMA. Additionally, the group differences in asymmetry score can find the reasons resulted from group-by-hemisphere interaction. When comparing the group differences in the asymmetry score, significant differences (*P* < 0.05, Bonferroni-corrected) were observed in the SMG, SOG, ROL, ITG, LING, IPL, ACG, and SMA. Specifically, patient with BD showed significant less rightward asymmetry in the ROL, SOG, and SMG, and more leftward asymmetry in the IPL, ACG, ITG, and LING, which were attributed to significantly more reductions nodal efficiency in right hemispheric. Only nodal efficiency of SMA in left hemisphere significantly reduced in patients with BD when compared with normal controls, and resulting in reversed hemispheric asymmetry.

### Relationship Between Hemispheric Asymmetry and BD Symptom Severity

**FIGURE 5:**
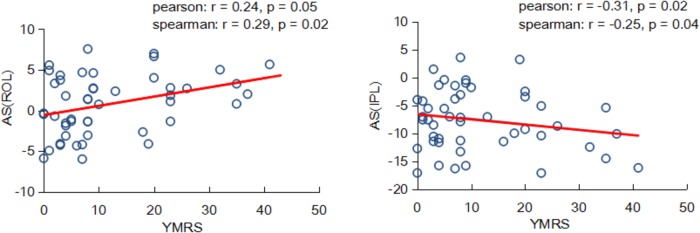
Partial correlation coefficients between the nodal asymmetry scores and clinical features of patients with BD.

Interestingly, we found the asymmetry scores of regional properties were correlated with BD symptom severity no matter using the Pearson or Spearman method. As shown in **Figure 5**, the nodal efficiency in ROL showed a significant and positive correlation with YMRS [AS(ROL), Pearson: *r* = 0.24, *P* = 0.05; Spearman: *r* = 0.29, *P* = 0.02], and the nodal efficiency in IPL showed a significant and negative correlation with YMRS [AS(IPL), Pearson: *r* = -0.31, *P* = 0.02; Spearman: *r* = -0.25, *P* = 0.04].

## Discussion

This current study employed DTI tractography and graph theory to examine the hemispheric asymmetries in brain WM networks in patients with BD. The hemispheric networks of patients were observed to have abnormal small-world property and reduced in global integration. Significant group-by-hemisphere interaction was revealed in the global efficiency, characteristic path length, and small-world property, which was attributed to significantly reduced global integration and increased small-word characteristic of right hemisphere in patients with BD compared with those in normal controls. Specifically, we found atypical asymmetric nodal efficiency in several regions mostly located at the parietal, temporal, and occipital areas. Furthermore, we revealed that the network properties were significantly correlated with symptom severity in BD. Our findings might provide a potential neural biomarkers of that the altered hemispheric asymmetries in brain anatomical networks for clinical presentation of BD.

### Hemisphere-Related Differences in Small-World Properties

In present work, hemispheric anatomical networks were used to evaluate the abnormal topological properties in patients with BD. Consistent with previous studies on whole-brain anatomical networks (Puetz et al., 2016; O’Donoghue et al., 2017), the hemispheric networks of both normal controls and BD patients preserved significant small-world property, that was significantly more clustered than random networks and had approximately the same characteristic path length as random networks (Watts and Strogatz, 1998). Moreover, we observed significant group differences in topological properties between normal controls and patients with BD. The smaller global efficiency, longer path length, and larger small-world property were shown in patients with BD compared with normal controls, suggesting a deficit in global integration (Munarini, 2013). Consistently, previous studies on whole-brain networks (Collin et al., 2016; Spielberg et al., 2016; Roberts et al., 2018) also showed smaller global efficiency and longer characteristic path length in patients with BD. Overall, these results suggested that the deficits in global integration were common in brain networks in patients with BD. Importantly, these abnormal global integrations were hemisphere-independent, which might be mainly due to the deficits of intra-hemispheric connections. Contemporary theories suggest that the complex clinical presentation of BD can be described as an aberration in the efficiency of information exchange between separate neural networks in the human brain (Vargas et al., 2013). Along this notion, our findings suggested the hemisphere-independent anatomical network with significantly smaller global efficiency and longer characteristic path length provides evidence for the hemispheric anatomical networks in patients with BD as a disconnection syndrome (Dae-Jin et al., 2013; Collin et al., 2016), especially for the right hemisphere.

The currently observed rightward asymmetry in global efficiency for normal controls suggests that the right hemisphere is intra-connected in a better integrated way, allowing for more efficient communication at the hemispheric level. Consistently, rightward asymmetry in network efficiency has been reported in healthy adults(Iturria-Medina et al., 2008; Sun et al., 2015). Interestingly, such rightward advantage in global integration in normal controls was absent in patients with BD. Instead, a roughly symmetrical pattern of global integration at the global level was found. The absence of rightward asymmetry in global integration was mainly due to the decrease of global integration in right hemisphere. According to lateralization of brain cognation theories, such absence of rightward asymmetry in global integration might underlie the BD dysfunctions in attention, visuospatial abilities (Cullen et al., 2016), and emotion regulation (Schwartz et al., 1975), which are considered to be dominantly processed in the right hemisphere. The decreased global integration in the right hemisphere was supported by the WM destruction in BD, specifically in the cingulum, corpus callosum, and superior longitudinal fasciculus (Ho et al., 2017). Our findings extend earlier work and provide network evidence that patients with BD have abnormal asymmetries in hemispheric networks and deficits in global integration in the functional networks of the right hemisphere.

### Hemisphere-Related Differences in Nodal Properties

Compared with normal controls, patients with BD exhibited reduced nodal efficiency in several regions (including the AMYG, the IPL, the PUT, and the TPOmid). Previous DTI studies (Rosso et al., 2007; Passarotti et al., 2012) also reported that patients with BD exhibited degenerated WM connectivity in these regions. For example, the disturbed anatomical connectivity (decreased FA) in the AMYG (Mcintosh et al., 2008) and TPOmid (Barnea-Goraly et al., 2009) may represent the reduction of nodal efficiency observed in this study. Haller et al. (2011) found that the gray matter of the PUT in patients with BD was significantly smaller than that in normal controls. The aberrations in nodal efficiency provided regional evidence to underline the neurobiological basis of BD.

In addition to the group differences in nodal efficiency, significant group-by-hemisphere interactions were found in ROL, SMG, ITG, SOG, and LING, attributing to significantly reduced hemispheric asymmetry in patients with BD compared with normal controls. The nodal efficiency of patients with BD showed more decreased in right hemisphere than left hemisphere relative to normal controls. Consistently, previous studies on BD revealed disrupted connectivity in the right hemisphere including ROL (Gernsbacher and Kaschak, 2003) and SMG (Wang et al., 2016), ITG, SOG, and LING (Green, 2006; Bearden et al., 2015). The right ROL and SMG regions were demonstrated involving emotional regulation (Silani et al., 2013). Considering one of significant symptoms of patients with BD was emotional regulation (Pavuluri et al., 2006, 2007), the reduction of nodal efficiency in these regions were proposed to associate with the impaired in emotional regulation in BD (Pavuluri et al., 2007). Moreover, it has been demonstrated that the right SOG, ITG, and LING regions were as associated with the visuospatial processing (Green, 2006; Bearden et al., 2015). The reduction of nodal efficiency in these occipital and temporal regions might be associated with the deficits in visuospatial functions (Green, 2006; Bearden et al., 2015).

Moreover, we also found that attention cognitive function regions including the ACG and IPL exhibited significant group-by-hemisphere interaction and group differences in asymmetry score. Because the nodal efficiency of right hemisphere in BD patients showed more reduction than those in left hemisphere, the ACG and IPL exhibited significant leftward hemispheric asymmetry. Consistently, previous studies have reported decreased connectivity in these two regions in patients with BD. For example, significantly decreased connectivity was found in the right ACG (Anand et al., 2009; Wang et al., 2009). Barnea-Goraly et al. (2009) reported that adolescents with BD had lower FA values than normal controls in the IPL region. Moreover, studies have showed that alterations in two regions are closely to the brain function in patients with BD. Patients with BD was demonstrated with decrease activation in the right ACG relative to normal controls during response inhibition task (Passarotti et al., 2010). The gray matter volume in the right parietal cortical regions correlated positively with the better inhibitory control in BD patients (Haldane et al., 2008). We further proposed that the abnormal asymmetries of nodal efficiency in patients with BD were associated with abnormal attention or inhibition function.

Interestingly, the patients with BD exhibited the significant rightward hemispheric asymmetry only in the SMA region, whereas a reversed asymmetry (leftward) in normal controls. The SMA region was revealed significant difference between the BD group and normal controls (Khadka et al., 2013). In addition, convergent studies (Caligiuri et al., 2003, 2004) have reported that patients with BD exhibited greater activities in right SMA during motor performance than those in left SMA. These results suggested that abnormalities in asymmetries of nodal efficiency in SMA influenced processing of motor function in BD (Puri et al., 2010; Rashid et al., 2014). These findings might provide potential neural biomarkers of the altered asymmetries in nodal efficiency for clinical presentation of BD.

### Relationship Between Regional Asymmetries and BD Symptom Severity

An interesting finding of the current study was that within patients with BD, the topological properties in hemispheric networks were related to BD symptom severity. We found that nodal efficiency was associated with the YMRS, which reflected the severity of the current manic episode; a higher YMRS resulted in a more severe episode. A positive relationship between the asymmetry score of nodal efficiency in the ROL and the YMRS was revealed, indicating that the more connectivity in the right ROL, the more severe symptoms of BD. Recently, Gao et al. (2017) found that connectivity in the right ROL was positively associated with the classification of BD. Moreover, a negative relationship between the asymmetry score of nodal efficiency in the IPL and YMRS was observed. We suggested that the negative correlation might result from significantly decreased connectivity in the right IPL. That is, the larger YMRS score the less connected communication in the right IPL region. Similarly, Barnea-Goraly et al. (2009) reported that adolescents with BD had lower FA values than normal subjects in inferior parietal region. Our observation suggested that abnormal properties of hemispheric asymmetries may underlie the dysfunctions existed in patients with BD.

## Conclusion

Using the DTI deterministic tractography method and graph theory, the current work evaluated the hemispheric effects on brain anatomical networks in patients with BD. The results revealed abnormalities in hemispheric asymmetries in patients with BD compared with those in normal controls. For the global network properties, the hemispheric asymmetry in global efficiency was significantly decreased and small-world property was significantly increased. Compared with normal controls, the nodal efficiency of patients also showed decreased rightward asymmetry mainly in the frontal, occipital, parietal, and temporal lobes. Exceptionally, the SMA region in patients with BD showed increased rightward asymmetries, attributing to a significantly reduction of the efficiency in left SMA. The asymmetry score of nodal efficiencies in the IPL and ROL exhibited correlations with clinical features of BD. These observations highlight that the altered hemispheric asymmetries in brain anatomical networks and the potential of brain hemispheric network measures as neural biomarkers for clinical presentation of BD. Our findings suggested that abnormal asymmetries in properties of hemispheric networks may underlie the dysfunctions in emotion and attention in patients with BD.

The research of this paper still has some limitations. In this study, the subjects was chosen from one site with small number of BD patient, limiting the statistical results of hemispheric effects and may bring about the type I error on brain network and region alterations. In order to evaluate accurately alteration in hemispheric asymmetry in patients with BD, the subjects with large number or from multi-site will be considered in the future work. Additionally, studies have reported that both gender and age are potential factors linked to brain asymmetry. Hence, we will further examine gender and age effects on hemispheric asymmetries in brain anatomical networks in BD.

## Ethics Statement

After receiving a thorough explanation, all participants gave written informed consent according to the procedures approved by the Taiyuan University of Technology.

## Author Contributions

BW completed the entire study of the experiment and writing. TL, MZ, SZ, YN, and XW revised the manuscript. TY, RC, and JX provided advice and guidance. DL provided the research ideas.

## Conflict of Interest Statement

The authors declare that the research was conducted in the absence of any commercial or financial relationships that could be construed as a potential conflict of interest. The reviewer YC and handling Editor declared their shared affiliation, at the time of the review.

## Footnotes

1 https://www.openfmri.org/

2 http://www.nitrc.org/projects/gretna/
